# Intracranial squamous cell carcinoma arising in a cerebellopontine angle epidermoid cyst: A case report and literature review

**DOI:** 10.1097/MD.0000000000009423

**Published:** 2017-12-22

**Authors:** Tae Hoon Roh, Yong Sook Park, Yong Gou Park, Se Hoon Kim, Jong Hee Chang

**Affiliations:** a Yonsei University Graduate School; b Department of Neurosurgery, Ajou University Hospital, Suwon; c Department of Neurosurgery; d Department of Neurological Surgery, Chung-Ang University Yongsan Hospital; e Department of Pathology, Brain Tumor Center, Brain Research Institute, Yonsei University Health System, Seoul, Republic of Korea.

**Keywords:** brain neoplasms, cerebellopontine angle, epidermoid cyst, malignant transformation, squamous cell carcinoma

## Abstract

**Rationale:**

Most of the intracranial epidermoid cysts are benign, but malignant lesions are occasionally reported. These lesions appear as squamous cell carcinoma and carry a dismal prognosis. Here, we report a case of a primary intracranial squamous cell carcinoma arising in a cerebellopontine epidermoid cyst. The relevant literatures were also reviewed.

**Patient concerns:**

A 53-year-old woman presented with dizziness and diplopia 9 months in duration. Magnetic resonance imaging revealed an epidermoid cyst in the left cerebellopontine angle and prepontine region with a focal enhancing lesion on T1-weighted gadolinium-enhanced images.

**Diagnoses:**

Histopathologic diagnosis revealed squamous cell carcinoma on a background of epidermoid cyst. Imaging studies excluded metastases.

**Interventions:**

The tumor was removed subtotally through a lateral suboccipital approach. The patient received intensity modulated radiation therapy (6720 cGy total) postoperatively.

**Outcomes:**

The patient was free from recurrence of the tumor until 3 years after surgery, at which point she was lost to follow-up. The patient died 4 years after the surgery.

**Lessons:**

The epidermoid cyst may occasionally become malignant. Finding an area of enhancement through preoperative magnetic resonance imaging can help to make a correct diagnosis. Based on the review of previous reports, surgical removal followed by radiotherapy shows the best result to treat malignant epidermoid cysts.

## 1 Introduction

Intracranial epidermoid cysts are rare congenital lesions that account for 0.2% to 1.8% of brain tumors.^[1,2]^ They are slow-growing benign tumors, thought to arise from misplaced epithelium.^[3]^ They occasionally degenerate into squamous cell carcinoma (SCC), although this is extremely rare. We report the case of a woman with primary intracranial squamous cell carcinoma arising from an epidermoid cyst. The relevant literature for this rare disease is also reviewed.

## 2 Case report

### 2.1 History and examination

A 53-year-old woman presented with a 9-month history of dizziness and diplopic vision. She had no relevant past medical history. Ophthalmologic examination revealed lateral gaze palsy of the left eye, suggesting cranial nerve VI palsy. Cranial nerve examination revealed no other cranial nerve abnormalities other than left cranial nerve VI palsy. The patient was awake, alert, and oriented; motor, sensory and cerebellar functions were normal.

### 2.2 Imaging studies

A non-contrast computed tomography (CT) scan of the head revealed an extra-axial low-density mass in the left cerebellopontine angle cistern extending to the prepontine region. The density of the mass was similar to that of cerebrospinal fluid. The pons and the basilar artery were slightly deviated toward the right due to compression by the mass.

**Figure 1:**
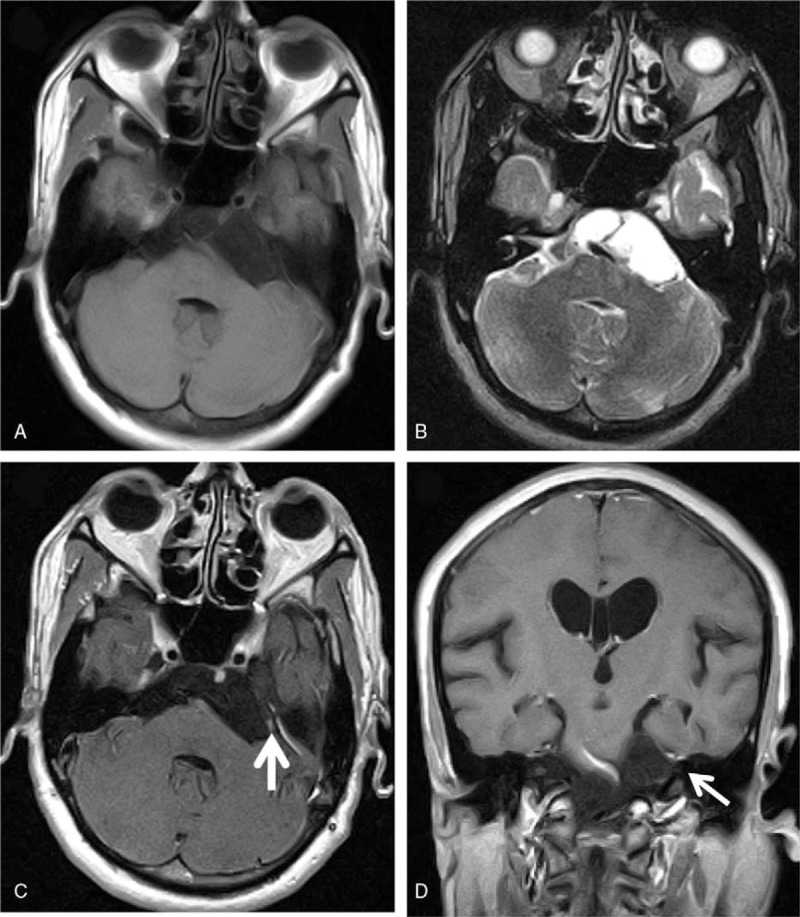
Preoperative MRIs show a cyst-like lesion in the left cerebellopontine and prepontine cistern on (A) T1-weighted axial image, and (B) T2-weighted axial image. On contrast enhanced (C) axial and (D) coronal images, the lesion shows a faint enhancement in its lateral portion (arrows). MRI = magnetic resonance imaging.

Magnetic resonance imaging (MRI) also revealed an extra-axial mass in the same region (Fig. 1). T1-weighted images without enhancement showed a low-intensity signal and T2-weighted images showed a high-intensity signal, which were consistent with an epidermoid cyst. There was no peritumoral edema; however, the T1-weighted images with enhancement by gadolinium-diethylene triamine pentaacetic acid showed a small, enhanced portion in the mass, which is not a typical feature of epidermoid cysts.

### 2.3 Surgical treatment and outcome

A left suboccipital craniotomy was performed and the tumor was gross totally resected. The tumor was pearly white, and appeared to have the typical gross appearance of an epidermoid cyst. Cranial nerves were identified and preserved.

Postoperatively, the patient had mild facial weakness, which improved gradually. Sixth cranial nerve palsy and hearing impairment remained the same.

One month after surgery, the patient began to receive intensity-modulated radiotherapy for a total dose of 67.2 Gy in 28 fractions. The patient tolerated the radiotherapy well.

### 2.4 Pathological findings

**Figure 2:**
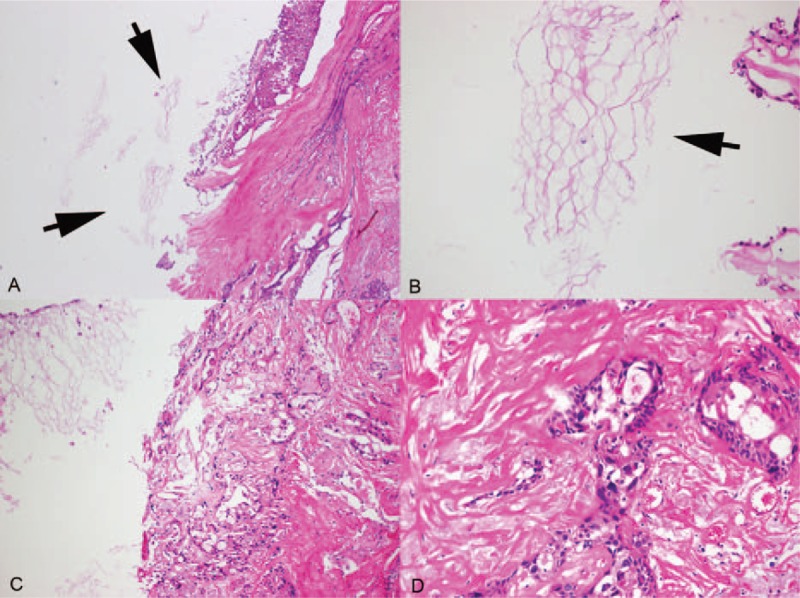
Pathologic findings of squamous cell carcinoma arising epidermoid cyst. Low power view showing thick fibrous tissue (right side) with scattered squames (arrows) (A: ×40, H-E) (B: ×200, H-E). In thick fibrous tissue, the carcinoma cell infiltration is noted (C: ×100, H-E). High power view showing definite infiltrating squamous cell carcinoma cells (D: ×200, H-E).

Histologic examination of the resected tumor specimen revealed the histological features of a poorly differentiated squamous cell carcinoma. However, in some portions, the tumor contained multiple layers of squamous epithelium lining and lamellar keratin, which are consistent with an epidermoid cyst (Fig. 2).

In order to rule out an extra-cranial primary tumor, abdominopelvic CT, chest CT, positron emission tomography (PET), and whole-body bone scan (WBBS) were performed, and all were normal. Given the histopathological features and the absence of extra-cranial malignancy, the SCC was thought to have arisen from a pre-existing epidermoid cyst.

### 2.5 Result of treatment

An MRI taken 3 months after the surgery showed a small enhancement in the left tentorium, which was thought to be a radiotherapy-induced change or a postoperative change. Nine months later, the enhanced volume had decreased. The 20-month follow-up MRI revealed that the enhanced portion had nearly disappeared, and showed no evidence of recurrence.

The patient's sixth nerve palsy had not improved after the radiotherapy, and the hearing impairment was aggravated, resulting in left ear deafness; facial weakness had resolved.

Forty-six months after the surgery, the patient was stable without any new symptoms, and MRI at last follow-up evidenced no recurrence. However, mild ventricular enlargement was suspected. The patient was lost to further follow-up, but was found to have died 4 years after surgery.

## 3 Methods

A systematic literature review was conducted using PubMed, Medline, and Google Scholar databases to search for relevant English language articles published up to March 2016. The following search terms were used: “malignant epidermoid cyst” OR “intracranial squamous cell carcinoma” OR “epidermoid cyst degeneration.” All eligible studies were reviewed, and the references were checked for additional relevant publications. Information and data were extracted from all included literature. Data include patients’ age, sex, treatment modality, overall survival, and recurrence-free survival. The ethical approval was not necessary, because this is a retrospective case report. The informed consent of the patient was given.

## 4 Results

We found 43 previously reported literature cases describing a malignant transformation from an epidermoid cyst (Table 1).^[4,5–46]^ There were 23 men and 21 women in these reports, with a mean age of 54 years (range, 4–74 years), including our patient. Their features do not differ from those of benign epidermoid cysts. Primary intracranial squamous cell carcinomas were present initially in 32 cases, while 12 cases were developed following an interval after the resection of a previous epidermoid cyst.^[8,10,25,27,30]^

**Table 1:**
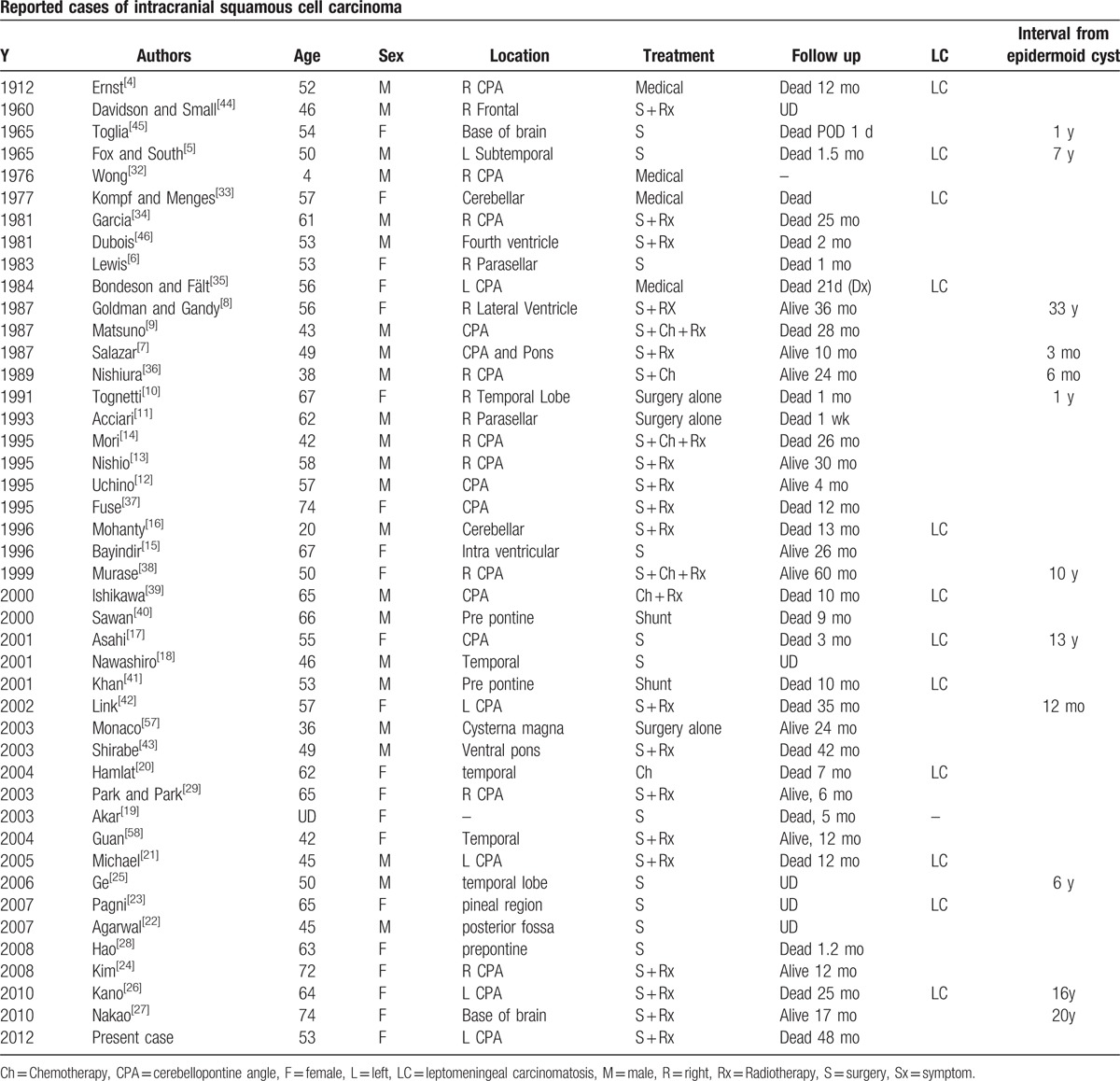
Reported cases of intracranial squamous cell carcinoma.

## 5 Discussion

Most squamous cell carcinomas involve the brain as a manifestation of metastases primarily originating from elsewhere in the body or as a result of direct invasion from head and neck cancer.^[47,48]^ Malignant transformation of an epidermoid cyst is extremely rare, but it has been well documented since Ernst^[4]^ first reported a case in 1912.

Garcia et al^[34]^ defined the criteria for such a malignant transformation as follows: the tumor had to be restricted to the intracranial, intradural compartment without invasion of or extension beyond the dura or cranial bones, and there must be no extension or invasion through intracranial orifices, no communication or connection with the middle ear, air sinuses, or sella turcica, and no evidence of nasopharyngeal tumor.

Hamlat et al^[49]^ reviewed 52 cases fulfilling Garcia's criteria and their additional criteria, as follows: presence of a benign squamous cell epithelium within the malignant tumor, and metastasis of carcinoma were excluded. Their review included SCC arising from other benign cysts rather than an epidermoid cyst, such as dermoid cysts, epithelial cysts, endodermal cysts, and craniopharyngiomas.

The present case fulfills Garcia and Hamlat criteria. No other malignancy found by systemic evaluation. Given that the epidermoid cyst was the only squamous epithelial tissue in the brain, we concluded that the squamous cell carcinoma had developed from a pre-existing epidermoid cyst.

The typical CT appearance of a benign intracranial epidermoid cyst is a low-density lesion in the subarachnoid space without contrast enhancement,^[50–53]^ although the margin of the cyst may exhibit minimal enhancement.^[7,54]^ An enhancement within an epidermoid cyst on CT or MRI suggests malignant transformation.^[12]^ Almost all of the cases we reviewed showed enhancement within the tumor, and, in some cases, malignant transformation of the enhanced portion was proven.^[6,13,16,46]^ In Kodama's case, the enhanced nodule was not resected during surgery and the pathological findings revealed an epidermoid cyst without malignant change^[31]^; however, a follow-up MRI 2 months after the surgery revealed growth of the enhanced region. The patient underwent stereotactic radiosurgery, but died 13 months after the first MRI evaluation. An autopsy revealed a poorly differentiated SCC originating from the epidermoid cyst. This case emphasizes that enhancement within an epidermoid cyst should be considered a sign of malignancy, and should be removed as possible.

Sudden development of symptoms or a growing epidermoid cyst seen on imaging study also suggests a malignant change.^[49]^ Epidermoid cysts grow linearly, thus, if they show exponential growth, malignant change should be considered.^[55]^

Diffusion-weighted MRI could be useful for the differential diagnosis of epidermoid cysts and SCC. A normal epidermoid cyst shows diffusion restriction on diffusion-weighted MRI, whereas transformed squamous cell carcinoma may not.^[18,31]^

The ideal treatment of epidermoid cysts is complete removal of the tumor and capsule^[2]^; however, this may result in unacceptable morbidity and mortality.^[7]^ The remnant tumor could lead to cyst recurrence or malignant transformation. If a patient has a remnant epidermoid cyst, follow-up to check for possible recurrence or a malignant transformation is necessary. The enhanced portion of the tumor should be removed to as great an extent as possible for accurate diagnosis and proper treatment. However, whether total resection of the tumor raises the survival rate compared with subtotal resection has not yet been investigated. Aggressive resection sometimes results in postoperative mortality.^[6,10,11]^

Postoperative radiotherapy has been used for the treatment of intracranial SCC and has been proven to improve short-term survival. Nagasawa et al^[56]^ reviewed 36 cases of malignant epidermoid tumors and compared survival outcomes between the surgery-alone group and the surgery plus radiotherapy group. Patients treated with surgery alone had an overall survival of 6.6 months, whereas those treated with postoperative radiotherapy demonstrated a statistically significant increase in survival of 12.7 months (log-rank test, *P* < .003). The mean dosage used during radiotherapy for the malignant epidermoids was 52.2 Gy. There was no significant correlation detected between the radiation dose and the survival outcome.

Radiosurgery can be used as an alternative or adjuvant to radiotherapy. There have been 4 reported cases in which stereotactic radiosurgery was applied to the treatment of malignant epidermoid cysts. Tamura et al^[30]^ performed a meta-analysis for the survival of 24 intracranial malignant epidermoid cysts. The median survival time for patients treated with surgery alone (n = 9), surgery plus external-beam radiation (n = 11), and surgery plus stereotactic radiosurgery (n = 4) were 1, 18, and 44 months, respectively (log-rank test, *P* < .004). However, this study was insufficient to advocate radiosurgery as a standard treatment. Because it was a retrospective study, there may have been selection bias, and the number of cases was small.

The prognosis of malignant epidermoid cyst is poor. Of the 43 cases of malignant epidermoid cysts we reviewed, 13 patients were reportedly alive, only 1 patient survived to 5-year follow-up, while 26 patients eventually died.^[38]^ All 11 patients with leptomeningeal carcinomatosis died.

The unique point of our patient is that the preoperative MRI showed the appearance of a typical epidermoid cyst, with only a small enhancement. Unless the possibility of a malignant epidermoid cyst has been considered in advance, it may not have been found in squamous cell carcinoma in pathologic results. An accurate diagnosis enabled adjuvant radiotherapy of this patient and resulted in relatively long survival period compared with the cases reported so far.

## 6 Conclusion

Malignant transformation of an epidermoid cyst is rare, and exhibits poor prognosis. The standard treatment for malignant epidermoid cyst is surgery and radiotherapy. Radiosurgery may replace or reinforce radiotherapy. Malignant transformation should be considered when the cyst exhibits contrast enhancement.

## References

[1] Ulrich J Intracranial epidermoids. A study on their distribution and spread. J Neurosurg 1964;21:1051–8. 14279825 10.3171/jns.1964.21.12.1051

[2] Schiefer TK Link MJ Epidermoids of the cerebellopontine angle: a 20-year experience. Surg Neurol 2008;70:584–90. 18423548 10.1016/j.surneu.2007.12.021

[3] Dias MS Walker ML The embryogenesis of complex dysraphic malformations: a disorder of gastrulation? Pediatr Neurosurg 1992;18:229–53. 1476931 10.1159/000120670

[4] Ernst P Haufung dysontogenetischer Bildungen am Zentral-nervensystems. Verhandl Dtsch Path Gesellsch 1912;15:226–30.

[5] Fox H South EA Squamous cell carcinoma developing in an intracranial epidermoid cyst (cholesteatoma). J Neurol Neurosurg Psychiatry 1965;28:276–81. 14345685 10.1136/jnnp.28.3.276 PMC495902

[6] Lewis AJ Cooper PW Kassel EE Squamous cell carcinoma arising in a suprasellar epidermoid cyst. Case report. J Neurosurg 1983;59:538–41. 6886769 10.3171/jns.1983.59.3.0538

[7] Salazar J Vaquero J Saucedo G Posterior fossa epidermoid cysts. Acta Neurochir (Wien) 1987;85:34–9. 3604769 10.1007/BF01402367

[8] Goldman SA Gandy SE Squamous cell carcinoma as a late complication of intracerebroventricular epidermoid cyst. Case report. J Neurosurg 1987;66:618–20. 3559730 10.3171/jns.1987.66.4.0618

[9] Matsuno A Shibui S Ochiai C [Primary intracranial epidermoid carcinoma accompanied with epidermoid cyst in the cerebellopontine angle--a case report]. No Shinkei Geka 1987;15:851–8. 3323934

[10] Tognetti F Lanzino G Manetto V Intracranial squamous cell carcinoma arising in remnant of extirpated epidermoid cyst. Br J Neurosurg 1991;5:303–5. 1892574 10.3109/02688699109005191

[11] Acciarri N Padovani R Foschini MP Intracranial squamous cell carcinoma arising in an epidermoid cyst. Br J Neurosurg 1993;7:565–9. 8267896 10.3109/02688699308995081

[12] Uchino A Hasuo K Matsumoto S Intracranial epidermoid carcinoma: CT and MRI. Neuroradiology 1995;37:155–8. 7761005 10.1007/BF00588635

[13] Nishio S Takeshita I Morioka T Primary intracranial squamous cell carcinomas: report of two cases. Neurosurgery 1995;37:329–32. 7477788 10.1227/00006123-199508000-00021

[14] Mori Y Suzuki Y Tanasawa T [A case report of epidermoid carcinoma in the cerebello-pontine angle]. No Shinkei Geka 1995;23:905–9. 7477700

[15] Bayindir C Balak N Karasu A Micro-invasive squamous cell carcinoma arising in a pre-existing intraventricular epidermoid cyst. Acta Neurochir (Wien) 1996;138:1008–12. 8891000 10.1007/BF01411292

[16] Mohanty VR Sastry Kolluri VSA Squamous cell carcinomatous change in a posterior fossa epidermoid: case report with a review of the literature. Br J Neurosurg 1996;10:493–6. 8922710 10.1080/02688699647140

[17] Asahi T Kurimoto M Endo S Malignant transformation of cerebello-pontine angle epidermoid. J Clin Neurosci 2001;8:572–4. 11683611 10.1054/jocn.2000.0856

[18] Nawashiro H Higo R Tokumaru AM Diffusion-weighted MRI of an intracranial epidermoid with malignant transformation. Neuroradiology 2001;43:891. 11688710 10.1007/s002340100612

[19] Akar Z Tanriover N Tuzgen S Surgical treatment of intracranial epidermoid tumors. Neurol Med Chir (Tokyo) 2003;43:275–80. discussion 281. 12870545 10.2176/nmc.43.275

[20] Hamlat A Hua ZF Saikali S Malignant transformation of intracranial epidermoid cyst with leptomeningeal carcinomatosis: case report. Acta Neurol Belg 2003;103:221–4. 15008508

[21] Michael LM 2nd Moss T Madhu T Malignant transformation of posterior fossa epidermoid cyst. Br J Neurosurg 2005;19:505–10. 16574566 10.1080/02688690500495356

[22] Agarwal S Rishi A Suri V Primary intracranial squamous cell carcinoma arising in an epidermoid cyst--a case report and review of literature. Clin Neurol Neurosurg 2007;109:888–91. 17826893 10.1016/j.clineuro.2007.07.026

[23] Pagni F Brenna A Leone BE Malignant epidermoid cyst of the pineal region with lumbar metastasis. Neuropathology 2007;27:566–9. 18021378 10.1111/j.1440-1789.2007.00820.x

[24] Kim MS Kim OL Primary intracranial squamous cell carcinoma in the brain stem with a cerebellopontine angle epidermoid cyst. J Korean Neurosurg Soc 2008;44:401–4. 19137089 10.3340/jkns.2008.44.6.401 PMC2615148

[25] Ge P Luo Y Fu S Recurrent epidermoid cyst with malignant transformation into squamous cell carcinoma. Neurol Med Chir (Tokyo) 2009;49:442–4. 19779295 10.2176/nmc.49.442

[26] Kano T Ikota H Kobayashi S Malignant transformation of an intracranial large epidermoid cyst with leptomeningeal carcinomatosis: case report. Neurol Med Chir (Tokyo) 2010;50:349–53. 20448435 10.2176/nmc.50.349

[27] Nakao Y Nonaka S Yamamoto T Malignant transformation 20 years after partial removal of intracranial epidermoid cyst--case report. Neurol Med Chir (Tokyo) 2010;50:236–9. 20339276 10.2176/nmc.50.236

[28] Hao S Tang J Wu Z Natural malignant transformation of an intracranial epidermoid cyst. J Formos Med Assoc 2010;109:390–6. 20497873 10.1016/S0929-6646(10)60068-X

[29] Park JW Park YM Primary intracranial epidermoid carcinoma. J Korean Neurosurg Soc 2003;34:159–61.

[30] Tamura K Aoyagi M Wakimoto H Malignant transformation eight years after removal of a benign epidermoid cyst: a case report. J Neurooncol 2006;79:67–72. 16583265 10.1007/s11060-005-9117-6

[31] Kodama H Maeda M Hirokawa Y MRI findings of malignant transformation of epidermoid cyst: case report. J Neurooncol 2007;82:171–4. 17004101 10.1007/s11060-006-9255-5

[32] Wong SW Ducker TB Powers JM Fulminating parapontine epidermoid carcinoma in a four-year-old boy. Cancer 1976;37:1525–31. 1260671 10.1002/1097-0142(197603)37:3<1525::aid-cncr2820370341>3.0.co;2-w

[33] Kömpf D Menges HW [Malignant degeneration in a parapontine epidermoid (author's transl)]. Acta Neurochir (Wien) 1977;39:81–90. 910653 10.1007/BF01405245

[34] Garcia CA McGarry PA Rodriguez F Primary intracranial squamous cell carcinoma of the right cerebellopontine angle. J Neurosurg 1981;54:824–8. 7017078 10.3171/jns.1981.54.6.0824

[35] Bondeson L Fält K Primary intracranial epidermoid carcinoma. Acta Cytol 1984;28:487–9. 6589931

[36] Nishiura I Koyama T Handa J Primary intracranial epidermoid carcinoma--case report. Neurol Med Chir (Tokyo) 1989;29:600–5. 2477760 10.2176/nmc.29.600

[37] Fuse T Takagi T Mizuno S [Primary intracranial malignant epidermoid--case report]. No To Shinkei 1995;47:997–1001. Japanese. 7577146

[38] Murase S Yamakawa H Ohkuma A Primary intracranial squamous cell carcinoma--case report. Neurol Med Chir (Tokyo) 1999;39:49–54. 10093462 10.2176/nmc.39.49

[39] Ishikawa S Yamazaki M Nakamura A [An autopsy case of primary cerebellar-pontine angle epidermoid carcinoma]. Rinsho Shinkeigaku 2000;40:243–8. Japanese. 10885335

[40] Sawan B Vital A Loiseau H Squamous cell carcinoma developing in an intracranial prepontine epidermoid cyst. Ann Pathol 2000;20:258–60. 10891726

[41] Khan RB Giri DD Rosenblum MK Leptomeningeal metastasis from an intracranial epidermoid cyst. Neurology 2001;56:1419–20. 11376208 10.1212/wnl.56.10.1419

[42] Link MJ Cohen PL Breneman JC Malignant squamous degeneration of a cerebellopontine angle epidermoid tumor. Case report. J Neurosurg 2002;97:1237–43. 12450053 10.3171/jns.2002.97.5.1237

[43] Shirabe T Fukuoka K Watanabe A Primary squamous cell carcinoma of the brain. A rare autopsy case. Neuropathology 2003;23:225–9. 14570292 10.1046/j.1440-1789.2003.00499.x

[44] Davidson SI Small JM Malignant change in an intracranial epidermoid. J Neurol Neurosurg Psychiatry 1960;23:176–8. 13814361 10.1136/jnnp.23.2.176 PMC495350

[45] Toglia JU Netsky MG Alexander E Jr Epithelial (epidermoid) tumors of the cranium. Their common nature and pathogenesis. J Neurosurg 1965;23:384–93. 5853888 10.3171/jns.1965.23.4.0384

[46] Dubois PJ Sage M Luther JS Case report. Malignant change in an intracranial epidermoid cyst. J Comput Assist Tomogr 1981;5:433–5. 6972395 10.1097/00004728-198106000-00025

[47] Bree RD Mehta DM Snow GB Intracranial metastases in patients with squamous cell carcinoma of the head and neck. Otolaryngol Head Neck Surg 2001;124:217–21. 11226960 10.1067/mhn.2001.112478

[48] Fowler BZ Crocker IR Johnstone PAS Perineural spread of cutaneous malignancy to the brain. Cancer 2005;103:2143–53. 15816051 10.1002/cncr.21004

[49] Hamlat A Hua ZF Saikali S Malignant transformation of intra-cranial epithelial cysts: systematic article review. J Neurooncol 2005;74:187–94. 16193391 10.1007/s11060-004-5175-4

[50] Davis KR Roberson GH Taveras JM Diagnosis of epidermoid tumor by computed tomography. Analysis and evaluation of findings. Radiology 1976;119:347–53. 1083544 10.1148/119.2.347

[51] Fein JM Lipow K Taati F Epidermoid tumor of the cerebellopontine angle: diagnostic value of computed tomographic metrizamide cisternography. Neurosurgery 1981;9:179–82. 6973704 10.1227/00006123-198108000-00014

[52] Gao PY Osborn AG Smirniotopoulos JG Radiologic-pathologic correlation. Epidermoid tumor of the cerebellopontine angle. AJNR Am J Neuroradiol 1992;13:863–72. 1590184 PMC8331691

[53] Latack JT Kartush JM Kemink JL Epidermoidomas of the cerebellopontine angle and temporal bone: CT and MR aspects. Radiology 1985;157:361–6. 3876574 10.1148/radiology.157.2.3876574

[54] Handa J Okamoto K Nakasu Y Computed tomography of intracranial epidermoid tumours with special reference to atypical features. Acta Neurochir (Wien) 1981;58:221–8. 7315553 10.1007/BF01407128

[55] Alvord EC Jr Growth rates of epidermoid tumors. Ann Neurol 1977;2:367–70. 617575 10.1002/ana.410020504

[56] Nagasawa D Yew A Spasic M Survival outcomes for radiotherapy treatment of epidermoid tumors with malignant transformation. J Clin Neurosci 2012;19:21–6. 22024232 10.1016/j.jocn.2011.06.002

[57] Monaco EA Friedlander RM Neuronal transplants reconstitute complex neuronal circuitry and rescue phenotype. Neurosurgery 2012;70:N11–2. 10.1227/01.neu.0000410931.31205.d2 22251979

[58] Guan LM Qi XX Zhang JR Intracranial squamous cell carcinoma developing in remnant of an epidermoid cyst: case report and literature review. Chin Med J (Engl) 2004;117:1880–3. 15603727

